# Bounded Graph Conditioning for LiDAR 3D Object Detection Under Sensor Degradation

**DOI:** 10.3390/s26092667

**Published:** 2026-04-25

**Authors:** Xiuping Li, Xiyan Sun, Jingjing Li, Yuanfa Ji, Wentao Fu

**Affiliations:** 1 School of Information and Communication, Guilin University of Electronic Technology, Guilin 541004, China; lxp863@gmail.com (X.L.); lijingjing@guet.edu.cn (J.L.); jiyuanfa@163.com (Y.J.); fuwentaoaa@163.com (W.F.); 2 Guangxi Key Laboratory of Precision Navigation Technology and Application, Guilin University of Electronic Technology, Guilin 541004, China; 3 National & Local Joint Engineering Research Center of Satellite Navigation Positioning and Location Service, Guilin University of Electronic Technology, Guilin 541004, China

**Keywords:** LiDAR sensing, 3D object detection, sensor degradation, graph conditioning, operating boundary, autonomous driving

## Abstract

Light Detection and Ranging (LiDAR) three-dimensional (3D) object detection degrades under point sparsity, outliers, coordinate noise, and calibration drift, yet detector evaluation remains largely limited to clean benchmarks. This study focuses on sensing robustness rather than detector redesign. We introduce Bounded Graph Conditioning (BGC)—a deterministic pre-voxelization front-end that applies *k*-nearest-neighbor (kNN) neighborhood averaging with bounded residual correction upstream of an unchanged detector backbone. BGC is evaluated together with a reproducible sensor-degradation stress protocol and a risk-constrained operating-boundary analysis. Experiments on KITTI with PointPillars, SECOND, and Voxel R-CNN show that BGC most clearly improves retained detection quality and feasible operating coverage under strong noise and strong outlier stress; gains under other degradation types are smaller and backbone-dependent. In the primary score-level box-disjoint calibration/test evaluation on SECOND, maximum feasible coverage at a target risk bound of 0.2 improves from 0.0754 to 0.1374 under strong noise (σ=0.10 m) and from 0.1323 to 0.1591 under strong outliers (p=0.10); a cross-backbone check on Voxel R-CNN confirms the same direction (0.1860→0.2864). Comparison with traditional filtering (SOR and ROR) reveals complementary strengths across fault types. A range-adaptive BGC variant that adjusts parameters per distance bin further improves performance under mixed unknown faults, spherical-coordinate noise, and on a dataset-matched nuScenes validation (adaptive BGC mAP/NDS: 0.2687/0.4493 vs. baseline 0.2471/0.3846 under strong noise). Severe translation drift collapses all configurations to full rejection, exposing an explicit sensing boundary beyond the reach of local conditioning. These results support BGC as a practical sensor-side robustness enhancement under the studied degradation protocol, with conditional rather than universal applicability across backbones and fault types.

## 1. Introduction

Light Detection and Ranging (LiDAR) is a core three-dimensional (3D) sensing modality in autonomous driving and robotics. In practice, however, performance failures in 3D object detection are often driven by degraded measurements—point sparsity, spurious returns, coordinate noise, and calibration drift—rather than by shortcomings in detector architecture on clean benchmarks [1,2,3,4,5].

Prior work typically addresses detector robustness, calibration, or confidence control in isolation. Sensor-facing deployment, by contrast, requires a combined view: how do degraded point clouds alter upstream feature formation, what clean-set cost does a robustness intervention incur, and when should the sensing channel be declared unusable rather than forced into overconfident prediction? This paper therefore asks a narrower question than end-to-end detector redesign: *can a lightweight deterministic front-end improve retained detection quality and feasible operating coverage under degraded sensing while preserving the downstream detector architecture?*

The main contributions are as follows.

1. **Reproducible sensor-degradation protocol.** We define a LiDAR stress protocol covering sparsity, outliers, coordinate noise, and calibration drift, with physically interpretable stress descriptors for systematic robustness reporting.
2. **Bounded Graph Conditioning (BGC).** We introduce a backbone-compatible, deterministic pre-voxelization front-end based on *k*-nearest-neighbor (kNN) neighborhood averaging with bounded residual correction, designed to limit upstream corruption amplification before an unchanged detector backbone. All reported results correspond to the same deterministic single-graph kNN implementation described in Section 4.
3. **Risk-constrained operating-boundary analysis with expanded cross-dataset validation.** We evaluate BGC over PointPillars [6], SECOND [7], and Voxel R-CNN [8] on the KITTI benchmark using degradation retention metrics, calibration summaries, and a risk-constrained acceptance analysis that delineates feasible and reject operating regions. An expanded dataset-matched validation on nuScenes [9] with the CBGS-SECOND-MultiHead detector provides additional large-scale evidence under a different sensor configuration. Quantitative comparison with traditional preprocessing methods (SOR and ROR), a systematic parameter sensitivity analysis, a mixed unknown-fault evaluation, and a spherical-coordinate noise proxy further characterize the applicability boundaries of the proposed front-end.

## 2. Related Work

### 2.1. LiDAR Sensor Degradation and Calibration Uncertainty

Stress testing and fault injection are standard tools for assessing degraded sensing under controlled conditions [10,11]. In LiDAR-based 3D object detection, practically relevant failure modes include point sparsity from adverse weather or weak returns, spurious ghost points, coordinate perturbations, and calibration mismatch from vibration or thermal shift [3,4,5]. Kim et al. [12] empirically quantified LiDAR detection performance degradation under real rain and fog on actual roads, showing that the number of returned point clouds and their intensity drop substantially with increasing precipitation and decreasing visibility. Broader evaluations of LiDAR perception algorithms under adverse weather—including point cloud filtering strategies for snow, rain, and synthetic fog—have further demonstrated that standard 3D detection and localization pipelines degrade significantly when sensor data quality deteriorates [13,14]. These studies motivate robustness evaluation at the sensing interface rather than solely at the detector-output level.

### 2.2. Robust 3D Object Detection and Risk-Aware Operation

Recent work on distribution shift, predictive uncertainty, and reliability-aware learning demonstrates that nominal detector accuracy is insufficient when confidence becomes unreliable under degraded inputs [15,16,17,18]. Voxel-based detectors such as SECOND [7] and Voxel R-CNN [8] and pillar-based encoders such as PointPillars [6] have been widely adopted for real-time LiDAR 3D object detection; Alaba and Ball [19] and Cai and Wu [20] provide recent surveys of deep-learning-based 3D object detection methods and their robustness-related characteristics across different LiDAR representations. To improve detection robustness against common LiDAR corruptions, recent approaches have explored diffusion-based point cloud purification as a preprocessing defense [21]. Compared with such generative purification methods, BGC offers lower computational cost, deterministic behavior, and no additional learned purification model, at the expense of a narrower correction scope limited to local neighborhoods. Risk control and selective prediction connect confidence calibration to accept/reject operation [22,23], but these approaches are typically applied as post hoc decision layers rather than as sensor-side conditioning mechanisms. This motivates studying how upstream point conditioning interacts with downstream confidence-aware acceptance under sensor degradation.

### 2.3. Graph-Based Preprocessing for Point-Cloud Sensing

Graph signal processing and polynomial graph filters offer interpretable local smoothing for irregular data [24,25]. Graph neural networks (GNNs) have been applied to point-cloud classification, segmentation, and detection [26]; in particular, Liang et al. [27] demonstrated that GNN-based 3D detectors can be accelerated by point-cloud preprocessing strategies that selectively preserve foreground structure while reducing computational load. Naich and Requena Carrión [28] proposed an intensity-aware voxel encoder for robust LiDAR 3D detection that leverages local point statistics within each voxel to improve resilience under adverse conditions. Relative to training-time robustness augmentation, generic denoising, or post hoc uncertainty scoring, our scope is narrower: a backbone-compatible front-end that modifies upstream point conditioning under sensor degradation while leaving the detector architecture unchanged. This positioning is deliberately sensor-facing rather than detector-redesign-oriented.

## 3. Problem Setting and Stress Protocol

### 3.1. Sensor-Degradation Taxonomy and Stress Grid

We model LiDAR sensor degradation and calibration mismatch as structured stress applied to the perception pipeline, following a stress–degradation view from controlled fault testing [10]. Severity is swept on a predefined grid and injected at inference time. Table 1 summarizes the four fault families—sparsity, outliers, Gaussian noise, and calibration drift—together with the corresponding severity grids.

A unified severity variable *s* is defined with monotonic direction “larger = more severe” across all fault families. For sparsity, severity is s=d=1−κ (with keep ratio κ retained for implementation and plot readability).

**Injection order and implementation.** For each frame, stressors are applied sequentially as follows: rigid calibration perturbation, additive noise, random dropping, then outlier insertion. Calibration drift is a rigid transform

(1) $$p^{\prime }=R_{z}\left(\theta \right)p+t,\;t={\left[\Delta x,0,0\right]}^{⊤},\;\theta =\mathrm{yaw}\_\deg\cdot \pi /180,$$

where yaw is specified in degrees (Table 1) and converted to radians for trigonometric operations. Point dropping uses uniform sampling without replacement with keep count ⌊κN⌋. Noise is independent and identically distributed (i.i.d.) isotropic Gaussian on Cartesian coordinates, $\epsilon ∼N(0,\sigma^{2}I_{3})$. Outliers are sampled uniformly in each backbone’s active point-cloud range: PointPillars uses x∈[0,69.12], y∈[−39.68,39.68], z∈[−3,1]; SECOND and Voxel R-CNN use x∈[0,70.4], y∈[−40,40], z∈[−3,1]. Extra channels are zero-initialized, with intensity sampled in [0, 1] when available. Random seeds are derived from frame IDs and a base seed for deterministic evaluation.

In engineering practice, these stressors approximate degradation modes such as packet loss or weak-return sparsity, spurious ghost points, coordinate perturbation, and extrinsic mis-calibration from vibration or thermal shift. The selected drift severities span nominal to clearly degraded regions, enabling localization of the onset of full rejection rather than probing small-perturbation robustness alone.

**Note on physical modeling.** LiDAR noise is often modeled in spherical coordinates (range and angles); we inject noise in Cartesian coordinates as a controlled approximation. The purpose is monotonic stress testing and cross-backbone comparability under a fixed protocol. A spherical-coordinate noise proxy experiment is reported in Section 6.9 for improved physical fidelity; extension to empirically calibrated weather-specific degradation models (rain, fog, and snow) remains future work.

### 3.2. Model-Agnostic Stress Descriptors

To complement task-level metrics, we report physically interpretable descriptors implied by the stress model. These descriptors are reporting aids rather than full physical observables. For point dropping with keep ratio κ, the expected sampling density scales as $\rho^{\prime }=\kappa \rho$. For Gaussian noise σ, the expected root-mean-square (RMS) positional perturbation is $\sqrt{3}\sigma$ in three Cartesian dimensions. For outlier ratio *p* injected after dropping, the final outlier fraction is approximately $\eta_{\mathrm{out}}\approx p/\left(1+p\right)$. For calibration drift (yaw θ, translation Δx), a point at range *r* experiences an approximate displacement

(2) δ(r,θ,Δx)≈2rsin(|θ|/2)+|Δx|,

which reduces to δ≈r|θ|+|Δx| for small angles. This linearized form is valid for $|\theta |<5^{\circ }$, covering the entire yaw grid in Table 1; at $\theta =1^{\circ }$ the relative error of the linear approximation is below 0.01%. These descriptors are used alongside average precision (AP), risk–coverage (RC), and calibration summaries in Section 6 to characterize the operational envelope. Table 2 summarizes notation used in subsequent metric definitions and result tables.

### 3.3. Robustness Metrics

Let m(s) denote an application-layer quality metric (moderate-difficulty AP on KITTI, R40 protocol) at severity *s*, and m(0) is the clean baseline. AP is treated as an *application-layer usability metric as follows*: it quantifies whether the stressed LiDAR input remains sufficiently informative for downstream perception, rather than directly measuring geometric sensor error. For all fault families, *s* is ordered in increasing severity; for sparsity, s=d=1−κ.

We summarize degradation trajectories using three complementary indices.

**Degradation–AUC ratio (D-AUC ratio).**

(3) $${\mathrm{AUC}}_{r}\;=\;\frac{\int_{s\in S}m\left(s\right)\;ds}{\int_{s\in S}m\left(0\right)\;ds},$$

computed via trapezoidal integration over the discrete severity grid (Table 1). Each fault family is normalized by its own stress range, so D-AUC is comparable within a fault type but not across different fault families.

**Worst-case performance ratio (WCPR).**

(4) $$\mathrm{WCPR}\;=\;\frac{\min_{s\in S}m\left(s\right)}{m(0)},$$

capturing weakest-condition behavior relevant to operating margins.

**Failure margin ($s_{\beta }$).**

(5) $$s_{\beta }\;=\;\max\left\{s\in S\;|\;m\left(s\right)\geq \beta \;m\left(0\right)\right\},$$

which estimates a tolerance boundary in stress space. We report β=0.8 (moderate margin, allowing a 20% performance drop) and β=0.95 (strict margin near nominal behavior). Let S denote the discrete severity grid for the degradation family under study. These thresholds serve as interpretable reference points.

### 3.4. Risk–Coverage Metrics for Operating Boundaries

We quantify confidence quality under sensor degradation using RC curves for risk-controlled acceptance [22], and we report the maximum feasible coverage $C^{⋆}\left(r^{⋆}\right)$ as MaxCov@0.2 when $r^{⋆}=0.2$.

Given a confidence threshold τ, we define the following:

(6) C(τ)=Pr(acceptedatτ),R(τ)=1−Precision(τ).

R and C are computed at the *box level* over all predicted boxes after non-maximum suppression (NMS) as follows: a prediction is accepted if its confidence score ≥τ. Precision is computed via greedy matching to ground-truth boxes (per frame, per class) using standard KITTI intersection-over-union (IoU) thresholds (Car 0.7, Pedestrian/Cyclist 0.5). C is the fraction of predicted boxes retained, aggregated across classes and frames. Threshold candidates with zero accepted boxes are treated as unavailable operating points.

RC curves are summarized by

(7) $$\mathrm{RC-AUC}=\int_{0}^{1}\left(1-R\left(C\right)\right)\;dC\approx \mathrm{trapz}\left(1-R_{i},C_{i}\right),$$

equivalent to the area under a precision–coverage curve (larger is better), and

(8) $$\mathrm{MaxCov}\left(r^{⋆}\right)=\max_{i}\left\{C_{i}|R_{i}\leq r^{⋆}\right\}.$$

Same-set summaries use 200-point subsampled RC curves for readability; the score-level box-disjoint calibration/test evaluation uses the full sorted calibration scores. This box-level definition yields a consistent risk proxy for erroneous accepted detections under sensor degradation, primarily controlling false-positive-side risk. False-negative-side behavior is tracked separately by the AP-based degradation summaries (D-AUC/WCPR/$s_{\beta }$). A system is operationally acceptable only if it achieves useful coverage while keeping risk below $r^{⋆}$ (we use $r^{⋆}=0.2$, i.e., ≥80% precision).

In the score-level box-disjoint protocol (Section 5), RC-AUC on the held-out test subset is computed by sweeping τ over the test-subset scores while keeping the threshold selection $\tau^{⋆}$ based on the calibration subset. This means the full RC curve shape is evaluated on the test partition, while only the operating-point selection depends on the calibration partition.

## 4. Method: Sensor-Side Graph Conditioning

**Figure 1:**
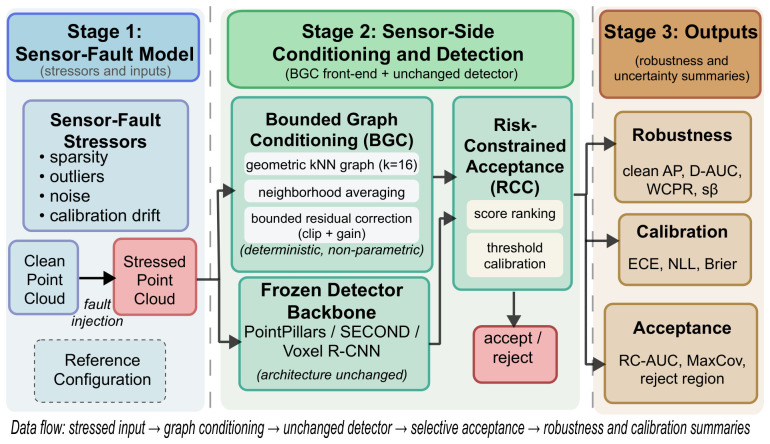
Pipeline overview. Stressors perturb the LiDAR input; the deterministic graph-conditioning front-end—Bounded Graph Conditioning (BGC)—performs kNN-based neighborhood averaging with bounded residual updates before an unchanged detector backbone (PointPillars, SECOND, or Voxel R-CNN); risk-constrained acceptance (RCC) maps confidence scores to feasible or reject regions under a target risk bound. Under high-outlier conditions, an optional reduced-gain variant (residual gain γ=0.5) replaces the default setting. The side channel denotes score and RC information used for threshold calibration. Colors group the three processing stages and their output summaries; arrows indicate data flow and threshold-calibration flow.

Figure 1 summarizes the graph-conditioning and operating-boundary assessment pipeline.

### 4.1. Deterministic Graph-Conditioning Front-End

The front-end is a deterministic graph-conditioning preprocessor applied before voxelization, not a learned detector submodule. We refer to it as *Bounded Graph Conditioning* (BGC) throughout.

Let $X\in R^{N\times d}$ denote the per-point feature matrix for one frame. The front-end updates coordinate and intensity channels only. A geometric kNN graph G is constructed on point coordinates with neighborhood size *k*. For each point *i*, denote its *k*-nearest neighbor set by $N_{k}\left(i\right)$. The neighborhood-averaged signal is computed as

(9) $$z_{i}\;=\;\frac{1}{|N_{k}\left(i\right)|}\sum_{j\in N_{k}\left(i\right)}x_{j},$$

where $x_{j}$ denotes the subvector of the coordinate and intensity channels of neighbor *j* that are updated by the front-end, and the summation uses uniform weights. The adjacency is binary (unweighted) and the neighborhood set $N_{k}\left(i\right)$ includes point *i* itself. We use k=16 for all KITTI experiments. This value provides a neighborhood radius that is large enough to suppress point-level noise while remaining small enough to avoid over-smoothing object boundaries at typical KITTI point densities; a systematic *k*-sensitivity study is provided in Section 6.8.

Let $Z\in R^{N\times d}$ collect all row vectors $z_{i}$. The front-end then applies a residual update with channel-wise clipping (Section 4.2).

**Placement and determinism.** The processor is applied before the voxel feature encoder (VFE), i.e., as point-level conditioning prior to voxelization, leaving the downstream detector unchanged. If the point cloud exceeds the configured maximum budget, random subsampling is used during training and a deterministic prefix subset during evaluation; updated points are scattered back into the original cloud. Graph construction and signal aggregation are deterministic given the input point set and evaluation seed.

### 4.2. Bounded-Residual Update

To convert neighborhood smoothing into an explicit bounded update, we impose

(10) $$X^{\prime }=X+\gamma \cdot \mathrm{clip}\left(Z-X;c\right),\;\gamma \in \left(0,1\right],\;c\in R_{+}^{d},$$

where clip(·;c) denotes elementwise clipping with per-channel bounds c. We use fixed bounds across faults and backbones ($c_{\mathrm{xyz}}=0.5\;m$ for spatial coordinates, $c_{\mathrm{int}}=0.3$ for intensity) and γ=1.0 for the default setting. The spatial clip bound $c_{\mathrm{xyz}}=0.5\;m$ is chosen to be well above the maximum noise standard deviation in the stress grid ($\sigma_{\max}=0.10\;m$) yet well below typical object extents (>1.5m for cars), thereby allowing meaningful correction without erasing object-level geometry. These bounds are held fixed to avoid stress-specific tuning. A reduced-gain sensitivity check is reported in Section 6.

**Bounded residual guarantee.** For any residual U=Z−X,

(11) $$∥X^{\prime }{-X∥}_{\infty }\leq \gamma {∥c∥}_{\infty },$$

where ${∥c∥}_{\infty }=\max\left(c_{\mathrm{xyz}},c_{\mathrm{int}}\right)$. This follows directly from the elementwise clipping in Equation (10), which caps each channel before the gain factor γ is applied. The update therefore remains bounded even when neighborhood aggregation is corrupted by extreme outliers.

**Controlled-sensitivity property.** Elementwise clipping is 1-Lipschitz as follows: ${∥\mathrm{clip}\left(U_{1};c\right)-\mathrm{clip}\left(U_{2};c\right)∥}_{p}\leq {∥U_{1}-U_{2}∥}_{p}$ for p∈{1,2,∞}. Equation (10) thus yields controlled sensitivity, preventing the unbounded amplification of corrupted neighborhood evidence. Because downstream backbones are not explicitly Lipschitz-constrained, Equation (11) provides an upstream bound rather than an end-to-end stability guarantee [29].

### 4.3. Reduced-Gain Variant for Severe Outliers

Outliers (ghost points) can poison local neighborhoods. The reduced-gain variant of BGC (denoted BGC-RG) applies a lower residual gain (γ=0.5) on the same deterministic front-end, limiting extreme residual updates under severe outlier contamination.

### 4.4. Range-Adaptive BGC Variant

LiDAR point density decreases rapidly with range, so a single set of conditioning parameters may over-smooth sparse far-range regions while under-correcting dense near-range areas. The range-adaptive BGC variant partitions the point cloud into range bins based on horizontal distance $r_{xy}=\sqrt{x^{2}+y^{2}}$ and applies bin-specific parameters. With range bins $\left[0,b_{1}\right),\left[b_{1},b_{2}\right),\left[b_{2},\infty \right)$ defined by boundaries $b=[b_{1},b_{2}]$, each bin *j* uses its own neighborhood size $k_{j}$, clip bounds $c_{\mathrm{xyz},j}$, $c_{\mathrm{int},j}$, and residual gain $\gamma_{j}$ as follows:

(12) $$X_{i}^{\prime }=X_{i}+\gamma_{b(i)}\cdot \mathrm{clip}\left(Z_{i}-X_{i};c_{b(i)}\right),$$

where b(i) denotes the range bin of point *i*. We use b=[20,40]m, k=[16,12,8], $c_{\mathrm{xyz}}=\left[0.5,0.3,0.1\right]\;m$, $c_{\mathrm{int}}=\left[0.3,0.2,0.1\right]$, and γ=[1.0,0.8,0.6] for near/mid/far bins, respectively. These values progressively reduce conditioning strength at longer ranges where sparser neighborhoods make aggressive smoothing counterproductive. The bounded-residual guarantee (Equation (11)) holds per bin with the corresponding bin-specific bounds.

### 4.5. Risk-Constrained Acceptance for Operating Boundaries

The risk-constrained acceptance rule (RCC) selects the threshold maximizing coverage under a risk constraint as follows:

(13) $$\tau^{⋆}=\arg\max_{\tau \in T(r^{⋆})}C\left(\tau \right),\;T\left(r^{⋆}\right)=\left\{\tau :R\left(\tau \right)\leq r^{⋆}\right\}.$$

In implementation, τ is searched over unique detector scores with nonzero accepted coverage.

**Infeasible region.** If $T(r^{⋆})=Ø$, no threshold satisfies the risk constraint. Maximum feasible coverage is then

(14) $$C^{⋆}\left(r^{⋆}\right)=\left\{\begin{array}{cc} \max_{\tau \in T(r^{⋆})}C\left(\tau \right), & T(r^{⋆})\neq Ø\\ 0, & T(r^{⋆})=Ø \end{array}\right.$$

and infeasibility constitutes an explicit reject decision rather than hazardous silent operation. We report *rejection* whenever $C^{⋆}\left(r^{⋆}\right)=0$, consistent with operational-envelope enforcement [30].

Same-set RCC summaries use discrete RC thresholds from score-ranked predictions; the score-level box-disjoint protocol calibrates $\tau^{⋆}$ on full calibration-score rankings and reports metrics on a disjoint subset. This is an empirical risk-constrained threshold-calibration rule; it does not provide conformal or finite-sample risk guarantees without additional calibration procedures.

## 5. Experimental Setup

We evaluate on KITTI [1,2] using the OpenPCDet toolbox [31]. The application task is 3D object detection; the validation emphasis is on sensing robustness, confidence calibration, and operating-boundary analysis under controlled stress. We employ the following three backbones: PointPillars [6], SECOND [7], and Voxel R-CNN [8]. We report the following: (i) clean AP (moderate-difficulty AP averaged over Car, Pedestrian, and Cyclist at KITTI R40), (ii) degradation metrics (D-AUC ratio, WCPR, $s_{\beta }$), (iii) calibration (ECE, NLL, and Brier score) [32], and (iv) RC summaries. Seed-0 plots illustrate representative curve shapes; key comparisons are supported by multi-seed summary tables.

**Training protocol.** For each backbone, we train both a baseline detector and a detector using the BGC front-end on the clean KITTI training split with backbone-specific schedules (PointPillars/SECOND: 80 epochs; Voxel R-CNN: 20 epochs). The front-end is deterministic and non-parametric; when enabled during training, the detector learns on conditioned point clouds. “Plug-in” refers to backbone-compatible insertion without redesigning the downstream architecture, not post hoc attachment to a separately pre-trained detector. No stress corruption is injected during training; all stress-grid evaluations use fixed checkpoints without stress-specific fine-tuning or online adaptation.

**Runtime.** End-to-end inference times are measured on a single reference platform (NVIDIA RTX A6000 48 GB, CUDA 12.1, PyTorch 2.3.1+cu121) as mean seconds per example over the full validation split (batch size: 2 for PointPillars/SECOND, 1 for Voxel R-CNN). All measured mean runtimes remain below a typical 10 Hz LiDAR scan period (100 ms). The overhead is dominated by kNN graph construction on the raw point cloud, not by the detector backbone itself; the current implementation uses a general-purpose kNN search without hardware-specific optimization, leaving room for further acceleration through spatial indexing or GPU-native neighbor search. The additional RCC computation is negligible because it only requires score sorting and threshold sweeping on stored outputs.

**Multi-seed aggregation.** Results are aggregated across seeds 0/1/2. Bootstrap confidence intervals (CIs) use N=10,000 resamples at 95% (α=0.05) [33]. These CIs are descriptive uncertainty bands, not hypothesis tests.

**Calibration, RC computation, and box-disjoint split protocol.** Calibration and RC are computed from detector outputs at each stress condition using greedy true-positive/false-positive (TP/FP) matching with KITTI IoU thresholds, 15-bin ECE, and score-ranked box-level RC curves aggregated across classes and frames. For same-set reporting, each RC curve is subsampled to 200 points. The score-level box-disjoint calibration/test split is performed as follows: after score extraction, box-level predictions are partitioned into calibration and test subsets (50/50); $\tau^{⋆}$ is selected on the calibration subset and RC-AUC/MaxCov/ECE are reported on the held-out test subset. Because the split is performed after score extraction at the box level, boxes originating from the same frame may appear in both subsets. The protocol is therefore a *score-level held-out threshold-calibration check* rather than a frame- or scene-disjoint deployment split; this distinction means that MaxCov values may be mildly optimistic relative to a fully frame-disjoint evaluation. An anonymous reproducibility package accompanies the submission.

## 6. Results

We organize results as follows: clean accuracy and robustness retention (Section 6.1), the primary box-disjoint operating-boundary evidence (Section 6.2), main-backbone ablation (Section 6.3), cross-backbone validation (Section 6.4), boundary-case analysis (Section 6.5), comparison with traditional preprocessing (Section 6.6), mixed unknown-fault evaluation (Section 6.7), parameter sensitivity analysis (Section 6.8), spherical-coordinate noise proxy (Section 6.9), and expanded cross-dataset validation on nuScenes (Section 6.10).

### 6.1. Accuracy, Robustness, and Runtime

**Figure 2:**
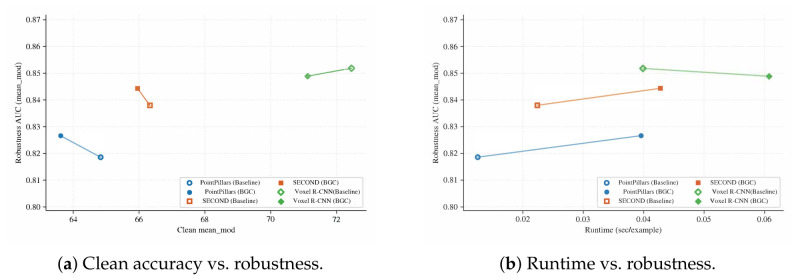
Pareto trade-offs between robustness retention (mean D-AUC ratio across fault families, vertical axis) and clean-set or runtime metrics (horizontal axis) for three detector backbones on KITTI val. (**a**) Clean moderate-difficulty AP (horizontal) vs. robustness retention; upper-right is preferred. (**b**) End-to-end runtime in sec/example (horizontal) vs. robustness retention; upper-left is preferred. Circles denote baselines; triangles denote the BGC front-end.

Figure 2 summarizes the trade-offs between robustness retention and (a) clean accuracy and (b) runtime. The BGC front-end is associated with higher robustness retention at a small clean AP cost, with moderate runtime overhead from graph construction (Table 3).

**Figure 3:**
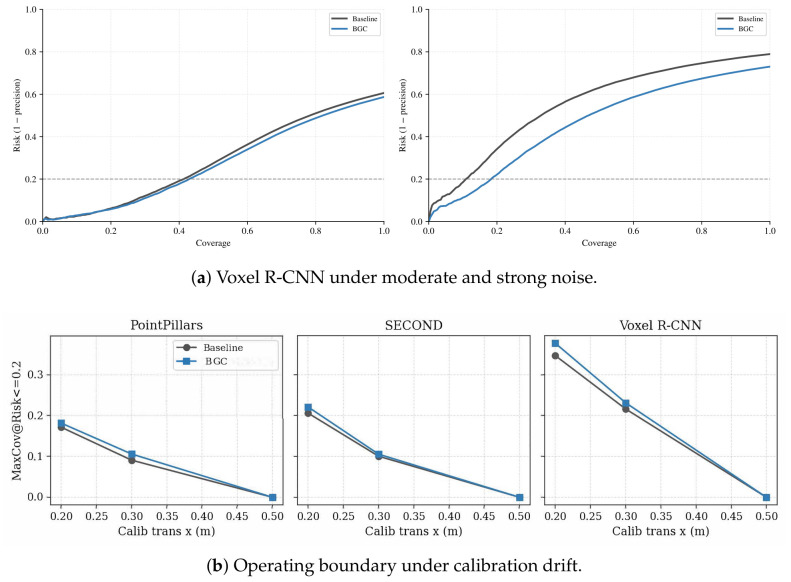
Operational summaries under sensor degradation (seed-0, same-set visualization). (**a**) Risk–coverage curves for Voxel R-CNN under moderate (σ=0.03) and strong (σ=0.10) Gaussian noise; the horizontal axis is coverage (fraction of accepted boxes) and the vertical axis is Risk (1−precision). (**b**) Maximum feasible coverage (MaxCov at $r^{⋆}=0.2$) as a function of calibration translation drift Δx for PointPillars, SECOND, and Voxel R-CNN; coverage drops to zero at severe drift, indicating full rejection.

Table 4 reports clean moderate-difficulty AP and per-fault D-AUC ratios. Table 5 provides bootstrap CIs for paired D-AUC/WCPR comparisons across seeds. D-AUC values are intended for within-fault comparisons (baseline vs. conditioned), not for ranking fault families against one another. Translation drift (Δx) is summarized by RCC operating-boundary metrics (Figure 3 and Appendix Table A2 and Table A3) because it behaves as structural misalignment with rapid feasibility collapse. Boldface marks strictly better values; ties are left unbolded.

Across PointPillars and SECOND, BGC increases retention under sparsity and moderate yaw drift with small clean AP reductions; Voxel R-CNN shows smaller gains and mild regressions on some stressors, indicating backbone-dependent trade-offs. These robustness summaries are computed before any RCC thresholding, thereby isolating the conditioning layer’s contribution. Additional multi-seed D-AUC summaries and calibration tables appear in Appendix A.

### 6.2. Primary Score-Level Box-Disjoint Operating-Boundary Evidence (SECOND)

The primary strong-fault evidence is the score-level box-disjoint calibration/test result in Table 6. For each seed and condition, score-ranked box-level predictions are partitioned into calibration and test subsets (50/50 split) after score extraction; $\tau^{⋆}$ is selected on the calibration subset only, and RC-AUC/MaxCov/ECE are reported on the held-out subset. This removes the threshold-selection optimism of same-set summaries.

Under box-disjoint evaluation, BGC improves RC-AUC/MaxCov from 0.3357/0.0754 to 0.4205/0.1374 for noise σ=0.10, and from 0.3616/0.1323 to 0.4092/0.1591 for outlier p=0.10. For translation drift Δx=0.5 m, both variants yield MaxCov=0, confirming the infeasible full-rejection regime and demonstrating that the analysis can identify explicit operating boundaries. The zero-variance baseline MaxCov (0.0754±0.0000) under strong noise indicates that all three seeds produce the same feasible-coverage ceiling at the discrete score-grid resolution; this is a consequence of the coarse threshold quantization at extreme degradation, not a data artifact.

Appendix Table A4 confirms that the method ranking is stable across $r^{⋆}\in \left\{0.1,0.2,0.3\right\}$, supporting $r^{⋆}=0.2$ as a representative working point. We also verified that a simple temperature-scaling baseline (Appendix Table A5) reduces ECE/NLL for both variants but leaves MaxCov unchanged, confirming that the front-end’s operating-boundary gains are not reproducible by post hoc score recalibration alone.

### 6.3. Main-Backbone Ablation (SECOND)

Table 7 isolates the conditioning-stage design on SECOND. Under strong noise, the main gain comes from adding graph conditioning, while bounded residual clipping provides a further marginal improvement (0.815→0.816 D-AUC) without materially changing clean AP. Under strong outliers, most of the gain already appears in the residual branch (0.972→0.980 D-AUC), with bounded clipping matching that level (0.979). Bounded clipping therefore serves as the default stabilizer for mixed faults and strong noise.

### 6.4. Cross-Backbone Validation and Operating Boundaries

Table 8 extends the box-disjoint protocol to Voxel R-CNN under σ=0.10. RC-AUC/MaxCov improve from 0.5362/0.1860 to 0.6325/0.2864, while ECE decreases from 0.3180 to 0.2793. This reduces the risk that the observed operating-boundary improvement is unique to SECOND.

Figure 3a visualizes how Voxel R-CNN RC changes between moderate and strong noise (same-set visualization; the primary quantitative evidence remains Table 6 and Table 8). From seed-0 outputs, noise σ=0.03 yields RC-AUC/MaxCov of 0.7140/0.4121 (baseline) vs. 0.7285/0.4271 (BGC); the strong-noise condition yields 0.4462/0.1055 vs. 0.5367/0.1809.

Figure 3b shows maximum feasible coverage as translation drift increases. SECOND MaxCov decreases from 0.2060 to 0.1005 to 0.0000 (baseline) and from 0.2211 to 0.1055 to 0.0000 (BGC); Voxel R-CNN shows the same collapse (0.3467→0.2161→0 baseline). At severe drift (Δx=0.5m), all evaluated backbones and methods are infeasible on all three seeds (infeasible rate =1.00). Tabulated diagnostics appear in Appendix Table A2 and Table A3.

**Figure 4:**
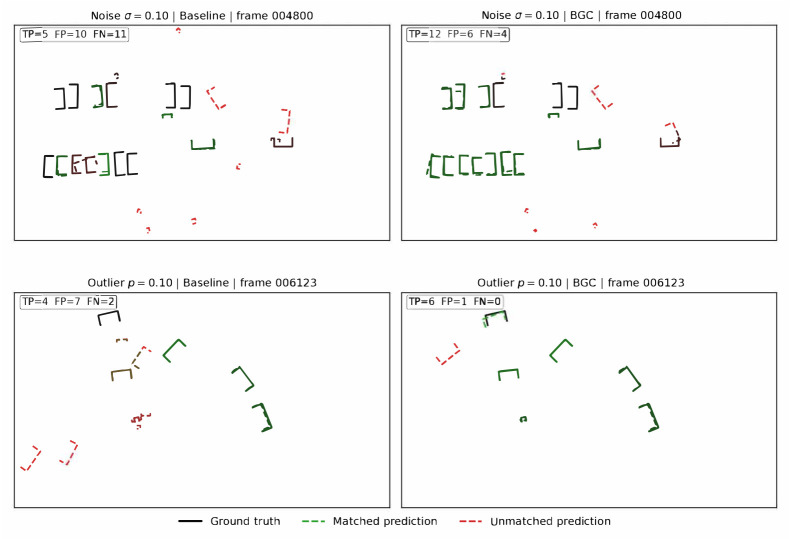
Qualitative detection comparison for SECOND on KITTI under strong noise (σ=0.10, top row, frame 004800) and strong outlier injection (p=0.10, bottom row, frame 006123). Left: baseline detector. Right: detector with BGC front-end. Black boxes: ground truth; green dashed: matched predictions (true positives); red dashed: unmatched predictions (false positives). BGC reduces false positives and recovers missed detections under both degradation modes. Frames were selected as representative of near-median detection performance under each fault condition.

Figure 4 provides a qualitative comparison of SECOND detection results under strong noise (σ=0.10) and strong outlier injection (p=0.10) on KITTI validation frames selected as representative of near-median detection performance under each degradation condition (neither best-case nor worst-case). Under noise, the baseline produces numerous false positives (FP=10) and misses most targets (FN=11), while BGC reduces false positives to 6 and increases true positives from 5 to 12. Under outlier contamination, the baseline generates 7 false positives with only 4 true detections; BGC suppresses false positives to 1 while recovering all 6 ground-truth objects. These frame-level examples illustrate the mechanism by which BGC improves the precision–recall trade-off under degraded sensing.

### 6.5. Boundary-Case Behavior Under Heavy Outliers

Table 9 provides a minimal parameter check for the reduced-gain variant (BGC-RG) on PointPillars under outlier stress (p∈{0,0.10}, single-seed). Across tested settings, gain and trim choices produce closely grouped D-AUC/WCPR values, indicating stable behavior across the tested hyperparameter range.

Table 10 compares the default BGC front-end with BGC-RG (γ=0.5) at p=0.10, showing improved tail risk (higher RC-AUC and MaxCov) for PointPillars and Voxel R-CNN under this condition. The default BGC front-end remains preferred for mixed-fault and noise-dominated conditions, while BGC-RG provides an effective alternative when outlier pollution dominates. This interpretation is consistent with the classical robust-statistics view that bounded influence and reduced sensitivity are beneficial under contamination-heavy regimes [34,35].

### 6.6. Comparison with Traditional Preprocessing Methods

To contextualize BGC against established point cloud filtering, Table 11 compares Baseline, BGC, Statistical Outlier Removal (SOR), and Radius Outlier Removal (ROR) on SECOND under strong noise and strong outlier conditions (same-set RC, seeds 0/1/2).

Under noise, BGC substantially outperforms both SOR and ROR, which achieve zero feasible coverage because they remove noisy points indiscriminately, discarding informative geometry. Under outlier contamination, ROR and SOR outperform BGC because explicit point removal directly targets the dominant fault. These complementary strengths motivate fault-adaptive mode selection (Section 7.4).

Table 12 reports runtime under both fault conditions. All methods remain below the 100 ms real-time constraint. Under the reported noise condition, the adaptive BGC variant is faster than fixed BGC because smaller *k* values in far-range bins reduce kNN search cost.

### 6.7. Mixed Unknown-Fault Evaluation

In deployment, the specific degradation state is unknown. Table 13 evaluates SECOND under a mixed unknown-fault protocol where each frame receives a random combination of faults (noise σ∈[0.02,0.10], outlier p∈[0.02,0.10], drop keep ratio ∈[0.7,1.0], and translation Δx∈[0,0.5]m) without fault-type labels provided to the method.

Fixed BGC with globally uniform parameters degrades overall mixed-fault utility because aggressive smoothing harms far-range detection as follows: Mean AP drops from 41.95 (baseline) to 26.24. The adaptive variant partially recovers the detection-side loss to 31.34 while more clearly improving operating-boundary quality as follows: RC-AUC improves from 0.3134 (baseline) to 0.3562, and MaxCov@0.2 from 0.0804 to 0.0955, with the largest gains in the near-range bin (<20 m). This mixed setting therefore highlights a trade-off rather than a universal gain as follows: adaptive conditioning improves the retained safe-operating region relative to both baseline and fixed BGC, but it does not restore the cleanest AP behavior of the baseline detector.

### 6.8. Parameter Sensitivity Analysis

Table 14 reports the sensitivity of BGC to neighborhood size *k*, clip bound $c_{\mathrm{xyz}}$, and residual gain γ on SECOND under strong noise (σ=0.10, seed 0), including distance-stratified RC (near <20 m, mid 20–40 m, far ≥40 m).

Increasing *k* monotonically degrades performance, especially at mid/far range where sparser neighborhoods cause over-smoothing. Tighter clip bounds ($c_{\mathrm{xyz}}=0.1$) yield the best overall metrics by constraining residual updates. Reduced gain (γ=0.5) also improves performance. The distance-stratified columns reveal a near–far trade-off as follows: settings optimal for near-range detection over-smooth far-range targets, directly motivating the range-adaptive variant (Section 4.4).

### 6.9. Spherical-Coordinate Noise Proxy

To improve physical fidelity beyond the Cartesian noise model, Table 15 reports SECOND under a spherical-coordinate noise proxy with range noise 0.15 m, azimuth jitter $0.30^{\circ }$, and elevation jitter $0.12^{\circ }$—values approximating realistic LiDAR noise characteristics where range uncertainty typically exceeds angular uncertainty.

Spherical noise is more destructive than isotropic Cartesian noise at comparable RMS levels because it couples range-dependent displacement with angular spread. The adaptive BGC variant approximately doubles mean AP relative to baseline (20.46→41.69) and substantially improves RC-AUC (0.3448→0.4864), confirming that range-adaptive conditioning is effective under physically motivated noise models.

### 6.10. Expanded nuScenes Validation Under Matched Training Conditions

To validate BGC beyond KITTI on a modern large-scale benchmark, we conduct an expanded evaluation on the nuScenes validation set [9] (6019 samples) using the CBGS-SECOND-MultiHead detector family [36]. Both the baseline and adaptive BGC detectors are trained under matched conditions from the official public checkpoint on the full nuScenes training split (28,130 samples), ensuring a fair comparison. Table 16 reports the mAP, NDS, and error metrics under clean and strong-noise (σ=0.10) conditions.

Under clean evaluation, adaptive BGC yields a small mAP gain (0.4835 → 0.4933), but it does not improve NDS or the reported error metrics. This indicates that, on nuScenes, the practical benefit of adaptive conditioning is concentrated under strong-noise degradation rather than clean inputs. Under strong Gaussian noise (σ=0.10), the gains become substantial as follows: adaptive BGC achieves mAP/NDS of 0.2687/0.4493, above the matched baseline (0.2471/0.3846) and the fixed BGC variant (0.2587/0.4210). All reported error metrics (mATE, mASE, mAOE, and mAVE) improve under noise, supporting the effectiveness of range-adaptive conditioning on a larger-scale, higher-density dataset when the training protocol is matched to the target sensor configuration. A full multi-backbone from-scratch nuScenes/Waymo benchmark is noted as future work (Section 7.5).

## 7. Discussion

### 7.1. Operating-Boundary Analysis Beyond Robustness Retention

While the front-end improves retained performance under stress, sensor-facing operation also requires a rule for when predictions should be trusted. RCC maps uncertainty to an operating boundary by enforcing $R\leq r^{⋆}$ and treating infeasibility as rejection rather than unsafe prediction. Under our definition, risk corresponds to the fraction of accepted predicted boxes that are false positives—a direct proxy for erroneous outputs that would propagate downstream. More broadly, sensor-driven perception must satisfy requirements beyond benchmark accuracy, including transparent operating regions and trustworthy behavior under degraded inputs [37]. The box-level RC framework presented here provides a tractable and consistent risk proxy; extending it to frame-level or scenario-level risk definitions is a natural direction for strengthening the connection to system-level safety requirements.

### 7.2. Why Bounded Gain Improves Robustness

The bounded residual update (Equation (11)) and 1-Lipschitz clipping prevent unbounded amplification of corrupted neighborhood evidence, providing a mechanism-level rationale for the observed robustness trends. This is an upstream bound rather than an end-to-end detector guarantee; it limits the damage from degraded neighborhoods without requiring changes to the detector architecture.

### 7.3. Structural Calibration Drift as a Limit of Local Conditioning

Translation drift is a global misalignment that local filtering cannot restore. RCC provides an explicit guard at boundary conditions where the perception channel is not safely usable, enabling fallback to alternative sensing, conservative speed control, or recalibration.

### 7.4. Conditional Operating Interpretation

The present results support a conditional operating interpretation rather than universal superiority across all backbones and degradation types. BGC most clearly improves retained utility and feasible operating coverage under strong noise and strong outlier stress, while gains under other settings are smaller and backbone-dependent; severe structural drift remains outside the capability of local conditioning. The comparison with SOR/ROR (Section 6.6) further reveals that BGC and classical point-removal filters have complementary strengths as follows: BGC excels under noise where point removal is counterproductive, while SOR/ROR excel under outlier contamination. When a fault monitor indicates heavy outlier contamination, the reduced-gain variant (BGC-RG) or classical outlier removal becomes a plausible alternative. When RCC finds no feasible threshold, the appropriate response is rejection and sensor-side fallback. The present evidence therefore supports BGC as a reasonable default mode under the studied mixed-fault protocol, with operating-mode adaptation as a practical engineering extension.

### 7.5. Scope and Future Directions

The present evidence has clear boundaries. The strongest score-level box-disjoint operating-boundary results are centered on SECOND, supplemented by one cross-backbone strong-noise check on Voxel R-CNN and a dataset-matched validation on nuScenes with the CBGS-SECOND-MultiHead family. The box-disjoint split operates at the score level and is not frame- or scene-disjoint; same-frame boxes may appear in both calibration and test subsets, which could lead to mild overestimation of MaxCov relative to a fully frame-disjoint split. The parameter sensitivity analysis (Section 6.8) covers k∈{8,16,24,32} and $c_{\mathrm{xyz}}\in \left\{0.1,0.3,0.5,0.7\right\}$; the range-adaptive variant addresses the near–far trade-off but its bin boundaries and per-bin parameters were selected empirically rather than optimized. The spherical-coordinate noise proxy (Section 6.9) improves physical fidelity over the Cartesian model, but extension to empirically calibrated weather-specific degradation data (rain, fog, and snow) remains future work. BGC cannot restore global structural drift. These factors mean the conclusions should be understood as conditional evidence under the studied degradation protocol rather than a universal robustness law.

The reproducible stress framework and evaluation methodology extend naturally to richer settings. A full multi-backbone from-scratch benchmark on nuScenes/Waymo, automated bin-boundary selection for the adaptive variant, and integration with fault-type classifiers for online mode switching between BGC and classical filters are natural engineering extensions. Frame-disjoint evaluation, frame-level risk definitions, and worst-case timing analysis would further strengthen operational relevance.

## 8. Conclusions

We presented Bounded Graph Conditioning (BGC)—a deterministic pre-voxelization front-end for LiDAR 3D object detection under sensor degradation—together with a reproducible stress protocol and a risk-constrained operating-boundary analysis. Robustness screening across three backbones on KITTI shows that BGC most clearly improves retained detection quality and feasible operating coverage under strong noise and strong outlier stress, with small clean-set cost; gains under other degradation types are smaller and backbone-dependent.

In the primary score-level box-disjoint evaluation on SECOND, RC-AUC/MaxCov improve from 0.3357/0.0754 to 0.4205/0.1374 for strong noise and from 0.3616/0.1323 to 0.4092/0.1591 for strong outliers; severe translation drift collapses to full rejection, exposing a clear operating boundary beyond the reach of local conditioning. A cross-backbone check on Voxel R-CNN under strong noise confirms the same direction (0.5362/0.1860→0.6325/0.2864). Comparison with traditional preprocessing (SOR and ROR) reveals complementary strengths across fault types: BGC excels under noise while SOR/ROR excel under outlier contamination. A range-adaptive BGC variant that adjusts parameters per distance bin improves performance under mixed unknown faults (RC-AUC: 0.3134→0.3562) and spherical-coordinate noise (mean AP: 20.46→41.69). On nuScenes under matched training conditions, adaptive BGC achieves mAP/NDS of 0.2687/0.4493 under strong noise, above the matched baseline (0.2471/0.3846), providing expanded large-scale validation evidence.

These results indicate that BGC can serve as a practical sensor-side front-end under the studied degradation protocol, with conditional rather than universal applicability. The accompanying stress framework provides a reproducible basis for further cross-dataset and cross-backbone evaluation.

## Figures and Tables

**Table 1:** LiDAR sensor-degradation taxonomy and severity grid. Larger severity uniformly denotes stronger stress across all families.

Fault Family	Stress Parameter	Severity Grid
Point dropping (sparsity)	drop ratio *d*; keep ratio κ=1−d	0.00, 0.25, 0.40, 0.50, 0.60, 0.75
Outlier injection	outlier ratio *p*	0.00, 0.01, 0.03, 0.05, 0.10
Gaussian noise	σ (m)	0.00, 0.01, 0.03, 0.05, 0.10
Calibration drift	yaw (deg); translation Δx (m)	yaw: 0, 0.2, 0.5, 1.0; trans: 0, 0.2, 0.5, 1.0

**Table 2:** Symbols and abbreviations used in the degradation and uncertainty analysis.

Symbol/Abbrev.	Meaning
*s*	Fault severity scalar (monotonic: larger = more severe; for sparsity, s=d=1−κ).
m(s)	Application-layer usability metric at severity *s* (moderate-difficulty AP on KITTI, R40 protocol, averaged over Car, Pedestrian, and Cyclist).
${\mathrm{AUC}}_{r}$	Degradation–AUC ratio (D-AUC ratio) over the stress grid.
WCPR	Worst-case performance ratio.
$s_{\beta }$	Stress margin where m(s)≥βm(0).
R(τ)	Risk at threshold τ (box-level 1−Precision).
C(τ)	Coverage at threshold τ (accepted fraction).
$r^{⋆}$	Target risk bound (set to 0.2 in experiments).
ECE	Expected calibration error (15-bin).
NLL	Negative log-likelihood.
Brier	Brier score.
RC-AUC	Area under (1−R) vs. coverage (precision–coverage AUC; higher is better).
MaxCov@0.2	Maximum coverage subject to R≤0.2.

**Table 3:** Per-example runtime on KITTI val (seed 0). Times are end-to-end means (sec/example) including graph construction, conditioning, and detector inference. Platform: RTX A6000, CUDA 12.1, PyTorch 2.3.1+cu121.

Backbone	Baseline (s)	+BGC (s)	Overhead (s)
PointPillars	0.0126	0.0396	+0.0270
SECOND	0.0225	0.0428	+0.0203
Voxel R-CNN	0.0399	0.0607	+0.0208

**Table 4:** Application accuracy and robustness summary on KITTI val (single-seed). D-AUC columns quantify performance retention; translation drift is summarized separately via RCC. Up arrows indicate that higher values are better. ΔAP denotes the clean-set cost of conditioning relative to the corresponding baseline. Boldface denotes the better value within each backbone/fault comparison block; ties are left unbolded.

Backbone	Method	Clean AP ↑	ΔAP	D-AUC(*d*) ↑	D-AUC(*p*) ↑	D-AUC(*σ*) ↑	D-AUC(yaw) ↑
PointPillars	Baseline	**64.826**	–	0.814	0.976	0.993	0.492
	+BGC	63.618	−1.208	**0.832**	0.976	0.993	**0.506**
SECOND	Baseline	**66.333**	–	0.885	0.983	**0.976**	0.507
	+BGC	65.959	−0.374	**0.901**	**0.988**	0.972	**0.516**
Voxel R-CNN	Baseline	**72.463**	–	0.917	**0.998**	**0.969**	**0.524**
	+BGC	71.125	−1.337	0.917	0.996	0.967	0.516

**Table 5:** Bootstrap 95% CIs for degradation indices (D-AUC/WCPR of moderate-difficulty AP) over seeds 0/1/2. Up arrows indicate that higher values are better. Δ denotes the difference between +BGC and baseline. Boldface denotes the better value within each paired comparison.

Backbone/Fault/Metric	Baseline [CI]	+BGC [CI]	Δ
PP/Outlier/D-AUC ↑	0.974 [0.971,0.976]	**0.978 [0.976,0.981]**	+0.005
PP/Outlier/WCPR ↑	0.954 [0.949,0.959]	**0.964 [0.962,0.967]**	+0.010
PP/Noise/D-AUC ↑	**0.992 [0.990,0.993]**	0.991 [0.989,0.993]	−0.001
PP/Noise/WCPR ↑	**0.961 [0.953,0.966]**	0.958 [0.955,0.959]	−0.003
SECOND/Drop/D-AUC ↑	0.896 [0.890,0.901]	**0.909 [0.907,0.911]**	+0.012
SECOND/Drop/WCPR ↑	0.635 [0.629,0.641]	**0.687 [0.678,0.695]**	+0.053
SECOND/Yaw/D-AUC ↑	0.507 [0.507,0.507]	**0.516 [0.516,0.516]**	+0.009
SECOND/Yaw/WCPR ↑	**0.082 [0.082,0.082]**	0.079 [0.079,0.079]	−0.003
VRCNN/Drop/D-AUC ↑	0.923 [0.922,0.925]	**0.926 [0.923,0.930]**	+0.003
VRCNN/Drop/WCPR ↑	0.709 [0.698,0.715]	**0.714 [0.710,0.719]**	+0.005
VRCNN/Yaw/D-AUC ↑	**0.524 [0.524,0.524]**	0.516 [0.516,0.516]	−0.008
VRCNN/Yaw/WCPR ↑	0.084 [0.084,0.084]	**0.086 [0.086,0.086]**	+0.002

PP = PointPillars; VRCNN = Voxel R-CNN.

**Table 6:** SECOND strong-fault score-level box-disjoint calibration/test evaluation (seeds 0/1/2, $r^{⋆}=0.2$). Thresholds are calibrated on a disjoint subset; metrics are evaluated on the held-out subset. Up/down arrows indicate that higher/lower values are better. Boldface denotes the better value within each paired comparison.

Condition	Model	RC-AUC ↑ (Test)	MaxCov@0.2 ↑ (Test)	ECE ↓ (Test)
Noise σ=0.10	SECOND baseline	0.3357±0.0031	0.0754±0.0000	0.1194±0.0007
Noise σ=0.10	SECOND + BGC	0.4205±0.0028	0.1374±0.0024	0.1222±0.0009
Outlier p=0.10	SECOND baseline	0.3616±0.0037	0.1323±0.0047	0.1329±0.0005
Outlier p=0.10	SECOND + BGC	0.4092±0.0019	0.1591±0.0024	0.1258±0.0006
Trans. Δx=0.5 m	SECOND baseline	0.2698±0.0020	0.0000±0.0000	0.2773±0.0009
Trans. Δx=0.5 m	SECOND + BGC	0.2916±0.0023	0.0000±0.0000	0.2717±0.0007

**Table 7:** SECOND ablation on the strongest corruption families (seed 0). BL = baseline; Res = residual-only (no clipping); ResClip = bounded residual (full BGC). Boldface denotes the better value within each row/metric comparison.

Fault	Clean AP			D-AUC			WCPR		
	BL	Res	ResClip	BL	Res	ResClip	BL	Res	ResClip
Noise	66.333	65.976	65.952	0.773	0.815	**0.816**	0.237	0.407	**0.407**
Outlier	66.331	65.982	65.951	0.972	**0.980**	0.979	0.950	**0.964**	0.963

**Table 8:** Voxel R-CNN strong-noise box-disjoint evaluation (seeds 0/1/2, $r^{⋆}=0.2$). Up/down arrows indicate that higher/lower values are better. Boldface denotes the better value within each paired comparison.

Item	Baseline	+BGC
RC-AUC (test) ↑	0.5362±0.0014	0.6325±0.0032
MaxCov@0.2 (test) ↑	0.1860±0.0071	0.2864±0.0082
ECE (test) ↓	0.3180±0.0007	0.2793±0.0021

**Table 9:** PointPillars outlier sensitivity for the reduced-gain variant BGC-RG (single-seed, p∈{0,0.10}). Configurations A–D vary gain γ and neighbor trim fraction *q*. Up arrows indicate that higher values are better.

Config	Gain *γ*	Trim *q*	D-AUC(*p*) ↑	WCPR(*p*) ↑
A	0.3	0.00	0.963	0.926
B	0.3	0.25	0.963	0.926
C	0.5	0.00	0.962	0.925
D	0.5	0.25	0.963	0.926

**Table 10:** Reduced-gain conditioning (BGC-RG, γ=0.5) under outlier stress (p=0.10, seeds 0/1/2, $r^{⋆}=0.2$). PP = PointPillars; VRCNN = Voxel R-CNN. Up/down arrows indicate that higher/lower values are better. Boldface denotes the better value within each paired comparison.

Condition	Model	RC-AUC ↑	MaxCov@0.2 ↑	ECE ↓
Outlier p=0.10	PP + BGC-RG	0.1738±0.0000	0.0452±0.0000	0.1388±0.0001
Outlier p=0.10	PP + BGC	0.1669±0.0002	0.0402±0.0000	0.1399±0.0001
Outlier p=0.10	VRCNN + BGC-RG	0.7533±0.0010	0.4874±0.0000	0.2567±0.0005
Outlier p=0.10	VRCNN + BGC	0.7492±0.0013	0.4791±0.0024	0.2599±0.0004

**Table 11:** SECOND comparison with traditional preprocessing methods (seeds 0/1/2, same-set RC). Up/down arrows indicate that higher/lower values are better. Boldface denotes the best value within each condition/metric comparison.

Condition	Method	RC-AUC ↑	MaxCov@0.2 ↑	ECE ↓
Noise σ=0.10	Baseline	0.2442±0.0020	0.0117±0.0024	0.1564±0.0003
	+BGC	0.3371±0.0010	0.0771±0.0024	0.1473±0.0005
	+SOR	0.1829±0.0027	0.0000±0.0000	0.1774±0.0001
	+ROR	0.1587±0.0011	0.0000±0.0000	0.1747±0.0003
Outlier p=0.10	Baseline	0.3247±0.0007	0.1072±0.0024	0.1470±0.0001
	+BGC	0.3727±0.0005	0.1323±0.0024	0.1382±0.0002
	+SOR	0.4502±0.0003	0.1776±0.0024	0.1507±0.0007
	+ROR	0.4988±0.0005	0.2111±0.0000	0.1528±0.0004

**Table 12:** SECOND runtime comparison across preprocessing methods (seed 0).

Condition	Method	Sec/Example	Avg Predicted Objects
Noise σ=0.10	Baseline	0.0166	16.452
	+SOR	0.0166	12.741
	+ROR	0.0429	17.375
	+Fixed BGC	0.0505	16.911
	+Adaptive BGC	0.0337	15.752
Outlier p=0.10	Baseline	0.0136	31.438
	+SOR	0.0168	19.639
	+ROR	0.0455	16.137
	+Fixed BGC	0.0478	26.138

**Table 13:** SECOND mixed unknown-fault evaluation (seed 0). Mean AP is the average of Car/Pedestrian/Cyclist moderate 3D AP_R40_ extracted from the corresponding KITTI evaluation logs; RC quantities are computed from the mixed-fault operating-boundary pipeline. Up/down arrows indicate that higher/lower values are better. Boldface denotes the best value among the compared methods for each metric.

Method	Mean AP ↑	RC-AUC ↑	MaxCov@0.2 ↑	ECE ↓	Near RC-AUC ↑
Baseline	41.95	0.3134	0.0804	0.1570	0.5062
Fixed BGC	26.24	0.3074	0.0804	0.1544	0.5548
Adaptive BGC	31.34	**0.3562**	**0.0955**	**0.1526**	**0.5646**

**Table 14:** SECOND parameter sensitivity under strong noise σ=0.10 (seed 0). Near/Mid/Far columns show distance-stratified RC-AUC. Up arrows indicate that higher values are better. Boldface denotes the best value in each metric column.

Setting	AP ↑	RC-AUC ↑	MaxCov ↑	Near ↑	Mid ↑	Far ↑
k=8,c=0.5,γ=1.0	32.22	0.363	0.106	0.611	0.358	0.045
k=16,c=0.5,γ=1.0	24.36	0.360	0.101	0.622	0.293	0.022
k=24,c=0.5,γ=1.0	18.79	0.345	0.091	0.610	0.235	0.011
k=32,c=0.5,γ=1.0	14.12	0.322	0.070	0.583	0.186	0.007
k=16,c=0.1,γ=1.0	**37.13**	**0.430**	**0.146**	0.634	**0.424**	**0.095**
k=16,c=0.3,γ=1.0	27.04	0.385	0.116	**0.635**	0.337	0.042
k=16,c=0.7,γ=1.0	23.96	0.354	0.101	0.620	0.284	0.021
k=16,c=0.5,γ=0.5	33.34	0.404	0.126	0.637	0.410	0.088

**Table 15:** SECOND under spherical-coordinate noise proxy (seed 0). Up/down arrows indicate that higher/lower values are better. Boldface denotes the best value among the compared methods for each metric.

Method	Mean AP ↑	RC-AUC ↑	MaxCov@0.2 ↑	ECE ↓	Near RC-AUC ↑
Baseline	20.46	0.3448	0.0854	**0.1366**	0.6853
Fixed BGC	22.83	0.3812	0.1156	0.1558	0.6770
Adaptive BGC	**41.69**	**0.4864**	**0.1859**	0.1471	**0.7372**

**Table 16:** nuScenes validation under matched training conditions with CBGS-SECOND-MultiHead. Both baseline and adaptive BGC use the same initialization and training protocol on the full nuScenes training split. Up/down arrows indicate that higher/lower values are better. Boldface denotes the best value within each condition/metric comparison.

Condition	Method	mAP ↑	NDS ↑	mATE ↓	mASE ↓	mAOE ↓	mAVE ↓
Clean	Baseline	0.4835	**0.6108**	**0.3138**	**0.2544**	**0.2327**	**0.3006**
	+Adaptive BGC	**0.4933**	0.6102	0.3170	0.2557	0.2451	0.3372
Noise σ=0.10	Baseline	0.2471	0.3846	0.4454	0.3962	0.4371	0.8298
	+Fixed BGC	0.2587	0.4210	0.4368	0.3434	0.4443	0.6476
	+Adaptive BGC	**0.2687**	**0.4493**	**0.3695**	**0.2701**	**0.3480**	**0.6527**

## Data Availability

The data presented in this study are based on publicly available benchmark resources and the OpenPCDet toolbox, which is available online at https://github.com/open-mmlab/OpenPCDet (accessed on 22 April 2026). Additional processed results supporting the conclusions of this article are available from the corresponding author upon reasonable request.

## Abbreviations

The following abbreviations are used in this manuscript:

LiDAR	Light Detection and Ranging
3D	Three-Dimensional
BGC	Bounded Graph Conditioning
BGC-RG	Bounded Graph Conditioning, Reduced-Gain variant (γ=0.5)
SOR	Statistical Outlier Removal
ROR	Radius Outlier Removal
AP	Average Precision
mAP	Mean Average Precision
NDS	nuScenes Detection Score
mATE	Mean Average Translation Error
mASE	Mean Average Scale Error
mAOE	Mean Average Orientation Error
mAVE	Mean Average Velocity Error
D-AUC	Degradation Area Under Curve
WCPR	Worst-Case Performance Ratio
ECE	Expected Calibration Error
NLL	Negative Log-Likelihood
RC	Risk–Coverage
RCC	Risk-Constrained Acceptance
kNN	*k*-Nearest Neighbors
NMS	Non-Maximum Suppression
IoU	Intersection over Union
VFE	Voxel Feature Encoder
TP	True Positive
FP	False Positive
FN	False Negative
CI	Confidence Interval
RMS	Root Mean Square
GNN	Graph Neural Network

## Appendix A. Supplementary Robustness, Calibration, and Operating-Boundary Results

This appendix provides supporting evidence including multi-seed robustness summaries, same-set RC results, calibration diagnostics, operating-mode comparisons, and temperature-scaling baselines referenced from the main text.

**Table A1:** Multi-seed D-AUC ratio (moderate-difficulty AP) for selected faults (seeds 0/1/2). BL = baseline. Up arrows indicate that higher values are better. Δ denotes the difference between +BGC and baseline. Boldface denotes the better value within each paired comparison.

Pair	BL D-AUC ↑	+BGC D-AUC ↑	Δ
PointPillars/Outlier	0.974±0.002	0.978±0.002	+0.005
PointPillars/Noise	0.992±0.001	0.991±0.002	−0.001
SECOND/Drop	0.896±0.004	0.909±0.002	+0.012
SECOND/Yaw	0.507±0.000	0.516±0.000	+0.009
Voxel R-CNN/Drop	0.923±0.001	0.926±0.003	+0.003
Voxel R-CNN/Yaw	0.524±0.000	0.516±0.000	−0.008

**Table A2:** RCC operating boundary at drift Δx=0.2 m ($r^{⋆}=0.2$, seeds 0/1/2). All methods feasible. BL = baseline. Up arrows indicate that higher values are better. Δ denotes the difference between +BGC and baseline. Boldface denotes the better value within each paired comparison.

Backbone	BL MaxCov ↑	+BGC MaxCov ↑	Δ
PointPillars	0.1709	**0.1809**	+0.0100
SECOND	0.2060	**0.2211**	+0.0151
Voxel R-CNN	0.3467	**0.3769**	+0.0302

**Table A3:** RCC operating boundary at drift Δx=0.3 m (seed 0, $r^{⋆}=0.2$). All rows feasible. MinRisk$\;=\;\min_{\tau }R\left(\tau \right)$. Up/down arrows indicate that higher/lower values are better, and boldface denotes the better value within each paired comparison.

Item	Baseline	+BGC
PP MaxCov ↑	0.0905	**0.1055**
PP Risk@Cov ↓	**0.1907**	0.1994
PP MinRisk ↓	0.0000	0.0000
SECOND MaxCov ↑	0.1005	**0.1055**
SECOND Risk@Cov ↓	**0.1970**	0.1981
SECOND MinRisk ↓	**0.0000**	0.1046
VRCNN MaxCov ↑	0.2161	**0.2312**
VRCNN Risk@Cov ↓	**0.1968**	0.1998
VRCNN MinRisk ↓	0.0000	0.0000

**Table A4:** MaxCov sensitivity to $r^{⋆}$ for SECOND strong faults (seeds 0/1/2).

Item	*r*^⋆^ = 0.1	*r*^⋆^ = 0.2	*r*^⋆^ = 0.3
Noise BL MaxCov ↑	0.0000±0.0000	0.0117±0.0024	0.0469±0.0024
Noise + BGC ↑	0.0151±0.0041	0.0771±0.0024	0.1189±0.0024
Outlier BL MaxCov ↑	0.0704±0.0000	0.1072±0.0024	0.1357±0.0000
Outlier + BGC ↑	0.0905±0.0000	0.1323±0.0024	0.1658±0.0000
Trans BL MaxCov ↑	0.0000±0.0000	0.0000±0.0000	0.0000±0.0000
Trans + BGC ↑	0.0000±0.0000	0.0000±0.0000	0.0000±0.0000

**Table A5:** Temperature-scaling baseline under SECOND strong-fault box-disjoint evaluation (seeds 0/1/2). ECE/NLL on held-out subsets (lower is better); MaxCov@0.2 for completeness. “raw” = without temperature scaling; “temp” = with temperature scaling.

Cond.	Model	ECE Raw ↓	ECE Temp ↓	NLL Raw ↓	NLL Temp ↓	MaxCov Raw ↑	MaxCov Temp ↑
Noise	BL	0.119±0.001	0.023±0.001	0.311±0.000	0.250±0.001	0.075±0.000	0.075±0.000
Noise	+BGC	0.122±0.001	0.021±0.001	0.300±0.001	0.230±0.001	0.137±0.002	0.137±0.002
Outlier	BL	0.133±0.001	0.045±0.001	0.220±0.001	0.130±0.001	0.132±0.005	0.132±0.005
Outlier	+BGC	0.126±0.001	0.039±0.001	0.228±0.001	0.142±0.001	0.159±0.002	0.159±0.002
Trans	BL	0.277±0.001	0.294±0.001	0.526±0.003	0.520±0.002	0.000±0.000	0.000±0.000
Trans	+BGC	0.272±0.001	0.285±0.001	0.516±0.002	0.511±0.001	0.000±0.000	0.000±0.000
